# Hair Cell Bundles: Flexoelectric Motors of the Inner Ear

**DOI:** 10.1371/journal.pone.0005201

**Published:** 2009-04-22

**Authors:** Kathryn D. Breneman, William E. Brownell, Richard D. Rabbitt

**Affiliations:** 1 Department of Bioengineering, University of Utah, Salt Lake City, Utah, United States of America; 2 Department of Otolaryngology - H&NS, Baylor College of Medicine, Houston, Texas, United States of America; 3 Marine Biological Laboratory, Woods Hole, Massachusetts, United States of America; Mount Sinai School of Medicine, United States of America

## Abstract

Microvilli (stereocilia) projecting from the apex of hair cells in the inner ear are actively motile structures that feed energy into the vibration of the inner ear and enhance sensitivity to sound. The biophysical mechanism underlying the hair bundle motor is unknown. In this study, we examined a membrane flexoelectric origin for active movements in stereocilia and conclude that it is likely to be an important contributor to mechanical power output by hair bundles. We formulated a realistic biophysical model of stereocilia incorporating stereocilia dimensions, the known flexoelectric coefficient of lipid membranes, mechanical compliance, and fluid drag. Electrical power enters the stereocilia through displacement sensitive ion channels and, due to the small diameter of stereocilia, is converted to useful mechanical power output by flexoelectricity. This motor augments molecular motors associated with the mechanosensitive apparatus itself that have been described previously. The model reveals stereocilia to be highly efficient and fast flexoelectric motors that capture the energy in the extracellular electro-chemical potential of the inner ear to generate mechanical power output. The power analysis provides an explanation for the correlation between stereocilia height and the tonotopic organization of hearing organs. Further, results suggest that flexoelectricity may be essential to the exquisite sensitivity and frequency selectivity of non-mammalian hearing organs at high auditory frequencies, and may contribute to the “cochlear amplifier” in mammals.

## Introduction

Hair cells of the inner ear are the primary mechanotransducers responsible for the sense of sound. At the apex of each of these cells are a bundle of 50–300 enlarged microvilli called stereocilia, the appearance of which earned the hair cell its name. The hearing organs from a variety of animals display a “tonotopic” gradation in the height of the hair bundles with shorter stereocilia located in the high-frequency sensing region of the organ and taller ones located in the low-frequency sensing region [1]–[3]. Here, we show that a flexoelectric motor mechanism provides a quantitative explanation for the observed tonotopic gradation in height in the cochlea.

Flexoelectricity is a term that was first coined to describe the orientation of liquid crystal molecules in the presence of an electric field. Later, membrane flexoelectricity (electricity that comes from flexing/bending) was hypothesized to play a role in biological membrane function [4]. Flexoelectricity manifests as a curvature induced electrical polarization of the membrane and, like piezoelectricity, can work in the forward direction to produce electrical polarization or in the reverse direction to produce changes in membrane curvature [5]. Petrov first proposed that forward flexoelectricity might underlie mechanotransduction in auditory hair cells by converting sound-induced changes in membrane curvature into displacement currents [6]. This observation is notable in that it recognizes the potential for large flexoelectric effects in hair-cell stereocilia membranes due to their small radii of curvature. The forward generator hypothesis, however, cannot explain the magnitude or temporal properties of the mechanoelectrical transduction (MET) current[7] and therefore does not underlie sensory transduction in hair cells, at least at frequencies studied to date. Here we examine the reverse hypothesis, that changes in membrane potential compel flexoelectric driven stereocilia movements. Motivating this hypothesis are recent data demonstrating that cylindrical membrane tethers with dimensions similar to hair cell stereocilia are electromotile and generate reduced tensile forces when depolarized [8]. These observations have led us to consider that stereocilia function as “flexoelectric motors”, taking electrical power entering the MET channels and converting it directly into mechanical power responsible for amplification of sound induced vibrations in the inner ear. Specifically, flexoelectricity endows the hair bundle with the ability to convert the displacement-sensitive MET current entering the tips of stereocilia into useful mechanical work, with the peak electrical to mechanical efficiency tuned to a best frequency dependent upon stereocilia length. We suggest that this mechanism is a key motor contributing to stereocilia bundle-based amplification and hearing sensitivity at high auditory frequencies [9].

**Figure 1:**
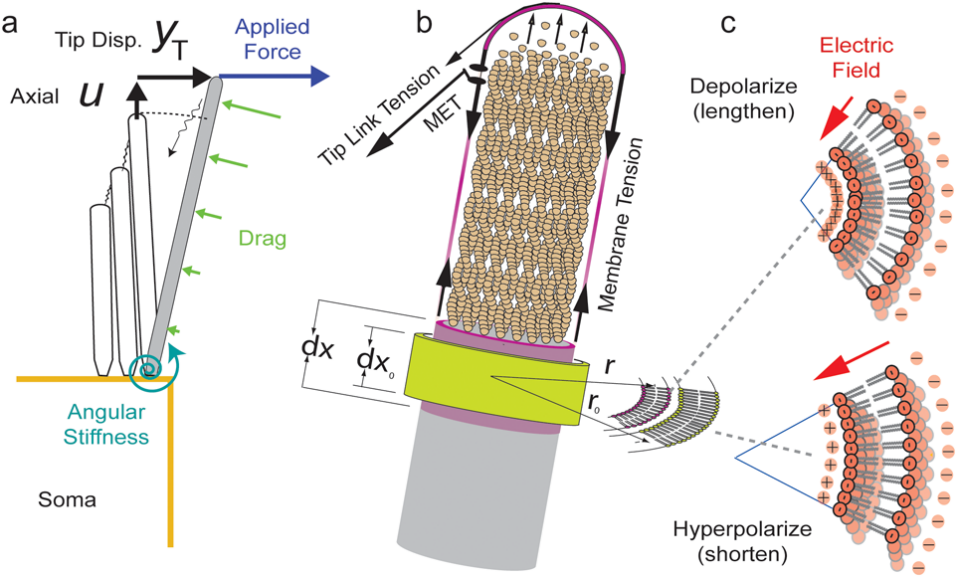
Stereocilium flexoelectric biophysics. a) As an excitatory force is applied the bundle deflects towards the tallest stereocilia and the tip link tension increases. Tip displacement causes the MET to open, current (*I_T_*) to enter the stereocilia, thus leading to cable-like membrane depolarization. b–c) Through the membrane flexoelectric effect, depolarization compels a decrease in radius (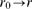) and increase in height (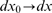) under constant volume. Changes in length are accompanied by transverse motion due to the staircase gradient in stereocilia lengths and diagonal tip links. Deflections are resisted by actin stiffness and polymerization at the tip, the angular stiffness at the base, and fluid drag in the axial and transverse directions.

To investigate flexoelectric power conversion, stereocilia were modeled as constant volume membranous cylinders with a filamentous elastic actin core. An excitatory force is applied causing deflection of the bundle towards the tallest stereocilia (Fig. 1a). Continuous polymerization of actin at the tip of the stereocilia generates the equilibrium force required to maintain the stereocilia height and, due to Newton's first law, provide a resting membrane tension (Fig. 1b). Since the two are coupled, modulation of stress and deformation in the membrane due to the flexoelectric effect, leads to modulation of stress and deformation in the actin core. Electrical depolarization of the membrane arises from displacement sensitive inward cation flow (Fig. 1c), and this compels a flexoelectric-generated increase in membrane curvature (decrease in radius) due to the interaction between the negatively charged polar lipid membrane heads and the transmembrane electric dipole [5]. Because of intracellular fluid volume conservation, this would compel a membrane surface area dependent lengthening of the stereocilia such that taller cilium will have a larger length increase than shorter structures. Conversely, during membrane hyperpolarization the curvature would decrease and the stereocilia would shorten. The stereocilia are arranged in a staircase architecture from short to long and are connected by angled tip links (Fig. 1b), therefore graded changes in length convert axial deformations into changes in tip-link force and lead to transverse motion of the bundle.

For maintained hair bundle displacements, the transduction current is known to adapt over multiple time courses due to kinetics of its molecular components. This electrical adaptation has a concomitant mechanical component that clearly contributes to active bundle movements [9]. Since flexoelectricity is downstream of the MET apparatus, the present analysis focuses on how flexoelectricity converts the current entering stereocilia, in whatever adapting temporal form it has, into useful mechanical work.

**Figure 2:**
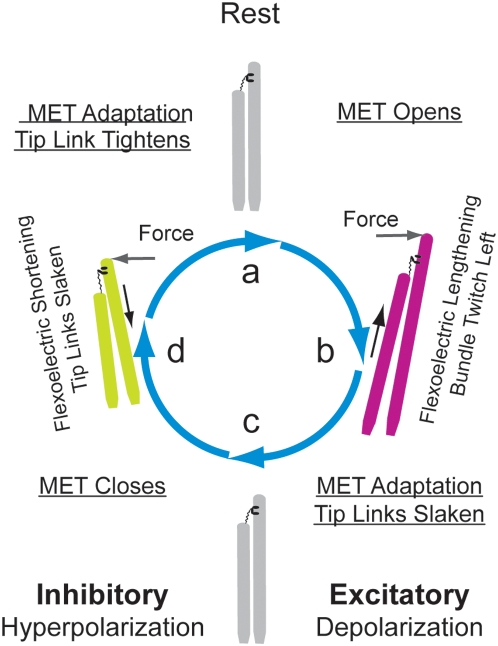
Flexoelectric Work Cycle. During excitatory stimulation, the bundle is pushed towards the tallest stereocilium causing opening of the MET channel and an influx of depolarizing current. b) Under these conditions, flexoelectricity compels an increase in the curvature (decrease in the radius) and an isochoric increase in length resulting in an increase in the tip-link tension and bundle movement towards the applied bundle force. This is accompanied by MET adaptation and associated nonlinearities. d) As the stimulus moves in the inhibitory direction, hyperpolarizing MET current causes decreased stereocilium curvature, axial shortening, tip-link slackening, and further relaxation of the bundle in the direction of applied force.

Under physiological conditions, sound stimuli entering the ear leads to forces that deflect the hair bundles from rest (Fig. 2a). As the bundle is pushed in the excitatory direction and the stereocilia are depolarized, flexoelectricity compels the radius to decrease (2b), length to increase, tip-link tension to increase, and finally a rapid bundle movement opposite in direction to that of the stimulation force. As the stimulus cycle progresses, the applied bundle force reduces to zero (2c) and then increases in the opposite, inhibitory direction producing hyperpolarization, a stereocilium radial increase, isovolumetric shortening (2d), and a further reduction in the tip-link tension that causes additional relaxation of the bundle in the inhibitory direction. Therefore, mechanical power provided by stereocilia flexoelectricity may interact with MET channel kinetics and nonlinearities to produce a limit cycle oscillation and amplify vibrations within the cochlea [10]. To investigate the feasibility of these ideas, we developed a relatively simple biophysical model to investigate power output of the flexoelectric mechanism (see Methods). Present results consider stereocilia in isolation from the MET channels by treating the MET current as a known input. Therefore results only address efficiency of the flexoelectric motor and do not address coupling to mechanical activation of MET channels or self-excited motion that would be expected to occur under some conditions.

## Results

**Figure 3:**
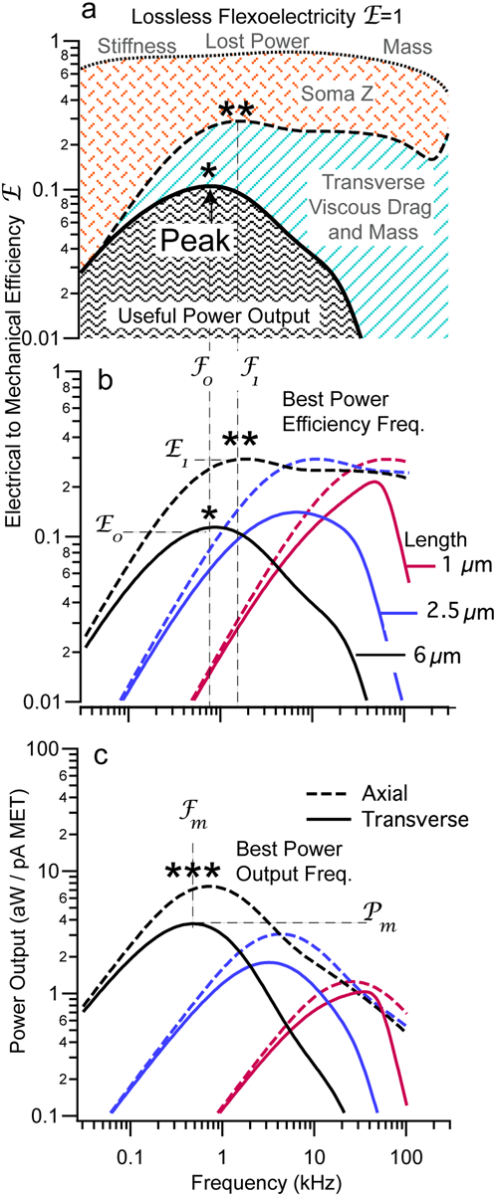
Power Efficiency. a) Taxonomy of power conversion for 6 µm long stereocilia showing peak efficiency of conversion at a specific best frequency (*). Input electrical MET power is lost to conductance of the soma and lost due to intrinsic mechanical properties of the stereocilia, including axial stiffness at low frequencies and entrained mass at high frequencies. Efficiency is further limited at high frequencies primarily by transverse viscous drag (light blue hatch). b) Peak conversion efficiency is tuned, with the optimum frequency (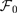, *) increasing as the stereocilia becomes shorter (3 lengths shown). Efficiencies are predicted to be higher for axial motion (dashed curves, 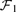, **) vs. transverse motion (solid curves, *). c) Power output is also predicted to be tuned with peak power occurring at a specific frequency (solid curves, 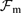, ***). Tuning is reduced if axial length changes are not coupled to cause transverse bundle motion (dashed curves).

**Figure 4:**
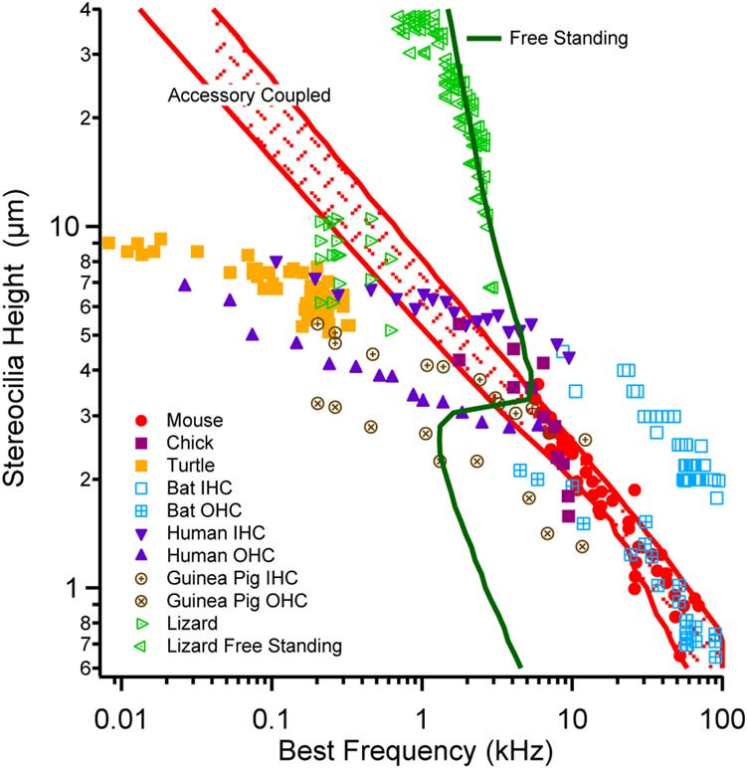
Universal phylogenetic law. Raw data (symbols) showing the height of the tallest stereocilia for cochlear hair cells from mouse [2], [44], human [2], [44], guinea pig [45], mustached bat [46], chick [47], alligator lizard [1], [13], [48] and the basilar papilla of turtle [49]. Flexoelectric model predictions show the frequency of peak efficiency for stereocilia of different heights that impart power to accessory structures (e.g. TM) but lose power to the fluid, and for freestanding stereocilia that impart power to the fluid through viscous pumping alone.

The efficiency of the electrical to mechanical conversion was estimated by dividing the output mechanical power by the input electrical power entering the stereocilia. In terms of efficiency, the flexoelectric model is linear so the overall magnitude of the power will be affected by the voltage and current changes but the calculated efficiency predictions will not. Efficiency predictions will be, however, affected by the degree of coupling between the stereocilia and accessory structures such as the tectorial membrane. Like skeletal muscle, the maximum power efficiency occurs for a load roughly half way between the zero-load condition and the maximum isometric force condition (termed the impedance matched load) [11]. Results shown in Fig. 3–4 assume an impedance-matched load, maximizing the transfer of power from the hair bundle motor to the dissipative cochlear load.

Shown for a ∼6 µm long stereocilia, broad-band power is lost to the somatic electrical admittance, intrinsic axial stiffness of the structure at low frequencies, transverse fluid drag in the mid-band, and entrained fluid mass at very high frequencies (Fig. 3a). The combination of these mechanisms results in a specific frequency for a given length stereocilia at which the electrical to mechanical power conversion is most efficient (Fig. 3a,b). Not surprisingly, the peak efficiency shifts to higher frequencies for shorter stereocilia. This tuning would be compromised if the MET channels were located uniformly along the length instead of at the stereocilia tips. In addition to the peak efficiency, the power output normalized to the input MET current was determined for a specific stereocilia length (Fig. 3c). The peak power output occurred at higher frequencies for shorter stereocilia while the magnitude of the output, not surprisingly, decreased with stereocilia height consistent with the decrease in membrane surface area available for electrical to mechanical power conversion. Of further interest, it can be seen that axial length changes when transversely coupled are more sharply tuned to a specific best frequency (solid line vs. dotted axial curves) thus indicating that the staircase architecture of hair bundles has a role in tuning as well.

Numerous studies have measured stereocilia height along the length of the cochlea. It is known in all auditory organs studied to date that each hair cell is associated with a neural “best frequency” at which the threshold for sound sensation is lowest. Maps have been composed for numerous species to correlate best frequency with location along the sensory epithelium. We combined data from multiple physiological and anatomical studies to plot the height of the stereocilia as a function of best frequency (Fig. 4) and found, with the exception of freestanding stereocilia discussed below, that across organs and species these data collapse to a simple relationship. For high-frequency hearing above ∼200 Hz, the relationship between stereocilia height observed in morphological studies and best physiological frequency has a slope of −1/2 (log-log), and for low-frequency hearing has a slope of −1/8. Above ∼200 Hz, the optimum stereocilia length predicted by the model (Fig. 4, red curve) reproduces the relationship between best frequency and stereocilium length appearing in nature. The red curve (TM coupled) was computed using the approach in Fig. 3 where we assumed power delivered to the fluid through viscous action along the shank was a lost and that the only useful power is extracted at the tip of the stereocilia by accessory structures such as the tectorial membrane (TM). In the case of freestanding stereocilium, there are no accessory structures attached to the tips and therefore any useful power output must be delivered directly to the fluid. Remarkably, by softening tip links and including power delivered to the fluid as useful mechanical output, the same model also predicts the relationship between best frequency and freestanding stereocilia length appearing in nature (Fig. 4, green curve). Results for freestanding stereocilium do not reflect a typical mechanical resonance balance between stiffness and mass[12], [13] but, instead, reflect a balance between stiffness, the flexoelectric effect, and axial electrical resistance. It is interesting that the bandwidth of freestanding stereocilia is quite narrow – this may be a key advantage of coupling hair bundles to a TM or similar accessory structure in hearing organs.

## Discussion

Below ∼200 Hz optimum stereocilium lengths predicted by flexoelectricity deviate from the lengths observed in nature (Fig. 3, −1/8 slope). Hence, if hair-bundle flexoelectricity were important at low frequencies, the motor would be inefficient. This suggests that other motor mechanisms associated with the MET molecular apparatus, such as unconventional myosin motors showing climbing and sliding rate limitations of 100 Hz and 44 Hz [14], respectively, or somatic motility[15] might have advantages at low frequencies. It is interesting that human hearing spans this range, as does hearing in many mammals including dogs, cats, guinea pigs and chinchillas. This opens the possibility that mammals may take advantage of one motor mechanism dominating at low frequencies and a different motor mechanism dominating at high frequencies. Present results show that stereocilia membrane flexoelectricity would be particularly tuned and efficient at high frequencies.

Support for the flexoelectric hypothesis also comes from genetic models of inherited hearing disorders. Flexoelectricity predicts that genetic models disrupting transverse connective links between adjacent stereocilia and/or disrupting the staircase ultrastructure of the bundle would result in impaired function of the cochlear amplifier. This is indeed the case. In adult myosin-XVa-deficient shaker 2 mice, the staircase architecture of hair bundles is lost and severe hearing loss occurs. Interestingly, these mice have nearly normal MET currents [16]. The present model predicts zero power output for these hair bundles because axial flexoelectric motion would not drive transverse deflection (see Eq. 10) and the power output would be zero. Similar results are found in stereocilin-deficient mice that lack horizontal top connectors, lateral links that connect adjacent stereocilia together [17]. The present analysis predicts hearing loss in both of these animal models due to disruption of the axial-transverse coupling normally exploited by the flexoelectric hair-bundle motor. There is evidence [18] suggesting that the tip-link insertion may not be near the top of the stereocilia, If this translates to the location of the MET current entering stereocilia, the primary effect would be to shorten the electrical path to the soma and thereby reduce the axial conductance. Such an arrangement would shift the most efficient frequency up slightly – by approximately 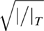, where 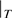 is the distance from the base to the MET channel and 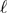 is the total length of the cilia.

Mechanical amplification of sound signals in the inner ear is controlled by the brain, in most species, through extensive efferent synaptic contacts on hair cells. In mammals, activation of the efferent system decreases mechanical amplification within the cochlea primarily through efferent action on outer hair cells [19]–[21]. A similar amplification control strategy is present in birds where efferent neurons contact short hair cells while afferents exclusively contact long sensory hair cells. The short hair cells in birds do not exhibit prestin dependent electromotility [22], but do have motile hair bundles thus implicating efferent innervation is controlling the hair bundle amplification in birds and other non-mammalian species. Control of the bundle motor by the efferent system presents a challenge to hypotheses that attribute cochlear amplification to the MET molecular apparatus because a clear mechanism for fast control *via* efferent synaptic input is unclear. In contrast, the power output of flexoelectric stereocilia described here is controlled by the electrical admittance of the hair cell soma, a parameter modulated by the efferent system [23]. In the present theoretical analysis, the power output at best frequency drops substantially when the somatic impedance is reduced. This occurs because the input MET power is lost to ground instead of being utilized to drive the flexoelectric hair bundle motor. Thus, hair bundle flexoelectric power output could be controlled by efferent modulation of somatic impedance.

It has been argued previously that active hair bundle movements may underlie the exquisite sensitivity and frequency selectivity of hearing, particularly in non-mammalian species that do not express prestin-mediated somatic motility [9]. Indeed, a negative bundle “twitch”[24] has been measured in hair bundles consistent with flexoelectric powered bundle movements (Fig. 2). Furthermore, the model predicts 200 aW of bundle power for a typical transduction current of 100 pA (2 aW/pA at 1 kHz), which compares favorably with a measured power output of 79 aW (79 zJ bundle work per cycle) [25]. In previous work, biophysics of the motor(s) has been closely associated with aspects of the MET complex[14], [26] but it has also been shown that voltage clamp of the hair cell soma evokes a very fast negative hair-bundle displacement even when the MET channels are completely blocked [27]. These voltage-dependent bundle movements augment motor actions associated with the MET apparatus and are consistent with the flexoelectric based bundle movements described here. Nevertheless, it has not yet been directly proven that flexoelectricity underlies the voltage-dependent responses in hair bundles and additional experiments will be necessary to test this hypothesis. The most direct experiments would involve investigations of axial force generation and/or membrane tension changes in individual stereocilia under somatic voltage clamp conditions with the MET channels blocked. Cholesterol and other compounds are known to influence the flexoelectric coefficient of membranes and thereby could be used to manipulate the force and displacement. Similar experiments could be done for transverse vs. axial motion comparing wild type to model organisms such as the myosin-XVa mutant lacking a staircase architecture. Manipulation of the actin core and protein accessory structure to modify axial and bending stiffness could also be revealing. Interestingly, the model suggests that as the cell is hyperpolarized, depending upon axial stiffness, there may be a critical voltage where the microvilli becomes unstable and suddenly bends in a way analogous to buckling of an axially loaded column.

Under physiological conditions, the flexoelectric motor would be powered by the MET current and thereby reflective of adaptations and temporal features of the MET molecular complex. Being independent of ATP and drawing from the large electro-chemical potential energy store of the inner ear endolymph fluid, the flexoelectric motor has great advantages of high speed and large power output over more conventional biological motors. Results suggest that the flexoelectric motor may generate the power-stroke of hair bundle motility (Fig. 2), at least at high frequencies above ∼200 Hz where ATPase would be too slow to operate on a cycle-by-cycle basis. Although our flexoelectric efficiency analysis is linear, interplay between MET current kinetics, bundle movements and flexoelectricity would be expected to introduce a nonlinearity consistent with spontaneous bundle oscillations. This interplay might underlie a limit cycle and Hopf[28] bifurcation that has been observed experimentally, and may be linked to the exquisite sensitivity of hearing [9]. Flexoelectricity also provides a simple explanation that, when thought of in terms of the efficiency of electrical to mechanical power conversion, proves adequate to predict the height of individual stereocilia as a function of cell best frequency and thus presents a universal explanation for the amazing tonotopic organization expressed in the cochlea.

## Methods

At rest, a stereocilia of length 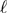 and radius *a* is in equilibrium with endogenous physical forces arising primarily from actin polymerization at the tip [29], MET tip link forces [30], membrane flexoelectricity [31], and passive mechanical forces [32]–[34]. In the present work we consider small perturbations from the equilibrium configuration leading to changes in axial force and length. The model does not attempt to describe the resting equilibrium configuration of stereocilia but only addressed dynamic perturbations from the equilibrium state. We model each stereocilia as a cylindrical lipid bilayer packed with actin filaments. The electro-mechanical equations follow directly from first principles of physics and can be reduced to an electrical cable equation coupled to a mechanical wave equation.

### Flexo-piezoelectric potential energy equivalency for axisymmetric, constant volume, deformations

In the present analysis we consider isochoric deformations such that for any stereocilia segment of differential length *dx* and radius *r* volume is conserved and we have: *πr^2^dx = πr_0_^2^ dx_0_*. This condition is expected to hold at auditory frequencies because of incompressibility of water and the low water permeability of the plasma membrane. The constant volume assumption was validated post-hoc by estimating the intra-stereocilia axial fluid flow per cycle (Poiseuille approximation) that would be driven by the flexoelectric perturbation in the intra-stereocilia pressure (Laplace approximation), and confirming that the axial flow is many orders of magnitude less than the stereocilia volume at frequencies addressed here. Therefore, a transmembrane electric field compelling a change in membrane curvature through the flexoelectric effect will compel a change in axial strain. This isochoric kinematic relationship allows flexoelectricity to be written in terms of the axial strain instead of a change in curvature. A simple way to find the axial equivalency is to equate the flexoelectric and axial piezoelectric electro-mechanical potential energies. The equivalency can be written [31], [35]

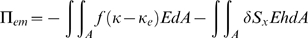
(1)where the integration is over the surface area, *A*, of the stereocilia membrane. The physical parameter, *f*, is the flexoelectric coefficient representing the strength of the coupling between the transverse electric charge displacement in the membrane and changes in the radius of curvature, *κ = 1/r*, relative to the equilibrium curvature *κ_c_ = 1/a*. The piezoelectric coefficient, *δ*, is the strength of the coupling between the transverse electric charge displacement in the membrane and the axial strain, *S_x_∼∂u/∂x*. The electric field acting across the membrane is *E = ν_/h_* where *v* is the local membrane potential and *h* is the membrane thickness. When the curvature is equal to the equilibrium curvature *κ_c_* (usually assumed to be zero), the membrane is in flexoelectric equilibrium and the flexoelectric energy is zero. The two terms in Eq. 1 are identical if 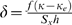. Under small deformations, the isochoric condition also requires dilation of the radius, radial strain *S_r_*, to be related to the axial strain *S_x_* by *S_r_ = −S_x_*/2. The change in curvature can be approximated for small constant volume deformations using a Taylor series expansion to find κ−κ_e_ = S_x_/(4a)+κ^*^. In this equation, *1/a* is the curvature of the stereocilia when in the resting reference configuration, and κ^*^. = (1/a)−κ_e_ is a constant relating the reference configuration to the flexoelectric equilibrium configuration. Using this, flexoelectricity can be represented in the piezoelectric electro-mechanical energy if we use the equivalent piezoelectric coefficient *δ∼f/(4ah)*. It is important to note that an increase in curvature (*κ>κ_e_*) corresponds to decrease in stereocilium radius (*r<a*) and, for the constant volume deformation, leads to a commensurate increase in axial length (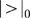) and increase in axial strain (*S_x_>0*).

The stiffness and mechanical potential energy arises from the actin core. In the present model we assume that the stereocilia height is maintained by polymerization if actin is at its tip and that this generates a resting tension in the membrane. By Newton's first law, the membrane tension is balanced by an equal but opposite resting compression in the actin bundle (Fig. 1). Since the membrane is fluid-like we do not expect that it would store any significant elastic potential energy. For simplicity, the model assumes that the axial strain in the core is equal to the axial strain in the membrane such that elastic deformation of the core directly gives rise to stress in the membrane.

### Electro-mechanical constitutive equations

Using the flexoelectric-piezoelectric equivalency condition for isochoric axisymmetric deformations allows us to study flexoelectric effects in stereocilia using the axial piezoelectric constitutive equations [36]:
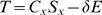
(2)and
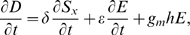
(3)where *T* is the axial stress in the membrane and *D* is the displacement current per unit membrane area, *C_x_* is the effective axial stiffness arising from the actin core, *δ = f/(4ah)* is the equivalent flexoelectric coefficient, *E* is the transmembrane electric field, *S_x_∼∂u/∂x*. is the axial strain, and ε is the membrane dielectric constant. We have augmented the standard equations with a membrane conductance *g* and associated conduction current. Integrating Eq. 6 around the circumference and thickness of the stereocilia membrane gives the axial force,
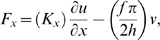
(4)where *K_x_ = πa^2^C_a_ = 2πahC_x_* (N) is the axial stiffness, *C_a_* (N/m^2^) is the actin core stiffness, and *C_x_* (N/m^2^) is the effective stiffness in Eq. 2. Under isometric conditions (zero strain, *S_x_ = 0*), the axial force generated in the stereocilia is proportional to the membrane potential and is *F_iso_ = −fπν/2h*. The negative sign shows that a positive tensile force will be generated when the intracellular voltage is negative relative to extracellular ground. The current per unit area through the stereocilium plasma membrane from Eq. 3 is
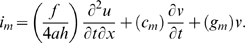
(5)Under isometric conditions (*S_x_ = 0*), this is the standard RC model with conductance, *g_m_*, and capacitance, *c_m_ = ε/h*, both per unit membrane area. Under zero load conditions (*F_x_ = 0*), the current reduces to
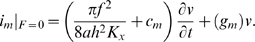
(6)The first term *πf^2^/8ah^2^K_x_* is the additional capacitance that arises from curvature induced charge movement. This increased capacitance during active motility is analogous to the voltage-dependent capacitance observed in the outer hair cell plasma membrane.

### Governing Equations

The cable equation was derived using the approach reviewed by Weiss [37], where the classical passive membrane current per unit area, 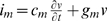 replaced with the flexoelectric version, Eq. 5, to find
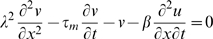
(7)where *v(x,t)* is the transmembrane voltage and *u(x,t)* is the axial displacement along the stereocilia, *t* is time, *x* is distance from the tip, *λ^2^* is the DC electrical space constant and *τ_m_* and is the classical passive membrane RC time constant. The MET current provides the electrical boundary condition at the tip and the hair cell somatic impedance provides the electrical boundary condition at the base. The flexoelectric parameter *β* is based directly on the known behavior of lipid bilayers and the geometry of the stereocilia.

The electro-mechanical wave equation was derived using the approach reviewed by Meirovitch 1967 [38], where the axial mechanical stress is replaced with the flexoelectric axial stress (Eq. 2) to find
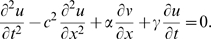
(8)where *c* is the passive mechanical wave speed, *γ* is a viscous damping coefficient for axial displacements, and 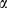 is the electro-mechanical coupling parameter (*α* is concomitant to *β* appearing in the cable equation) [39]. Specific relationships to the geometry and physical parameters are provided below.

Transverse deflections of stereocilium are modeled as linear, 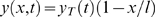, where the deflection at the tip, *x = 0*, is *y_T_(t)*. This motion is resisted primarily by viscous damping of the fluid, stiffness at the base, and to a lesser extent, mass (stereocilia and entrained water). The transverse motion is modeled as an equivalent mass, damping and stiffness *(m_T_,c_T_,k_T_)* lumped at the tip according to
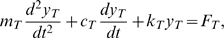
(9)where *F_T_* is the component of any applied force pushing the stereocilia in the excitatory direction plus the transverse component of the force from the tip link tension, *F_TL_*. The equivalent mass, damping and stiffness for the transverse motion were based on elastic properties of stereocilia[40] and Stokes flow using equations provided in Table 1. For stereocilium connected by tip links at angle 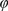, under small displacements
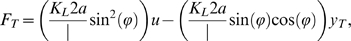
(10)where *K_L_* is the tip link stiffness. Eq. 7–9 were solved using an eigenfunction expansion as summarized below.

**Table 1:** Model coefficients and parameters.

*α = δ/ρh = f/4ρah^2^*	Wave equation flexoelectric coef. (m^2^/(V-s^2^))
*β = δ/g_m_ = f/4ahg_m_*	Cable equation flexoelectric coef. (V-s)
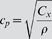	Passive mechanical wave speed (m/s)
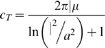	Stokes drag re: 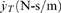 [50]
*δ = f/4ah*	Equivalent piezoelectric coef. (N/(V-m))
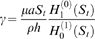	Fluid visco-elastic coefficient (s^−1^) [39]
*g_i_ = σ_i_A_i_ = 1/r_i_*	Axial conductance (S-m)
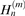	Hankel function of the *n^th^* order, *m^th^* kind
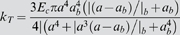	Transverse stiffness re: 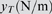 [40]
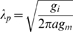	Passive electrical space constant (m) [51], [37]
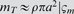	Transverse mass re: 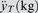 , ζ_m_ = 0.5
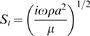	Complex Strouhal number (dimensionless)
*τ_p_ = ε /hg_m_ = c_m_/g_m_*	Passive membrane time constant (s)
*μ = μ_0_ (iω)^(ν−1)^*	Fluid viscoelastic modulus ( 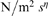 )

### Boundary conditions and general solution

The general analytical solution was written in the frequency domain as an eigenvector expansion[39]

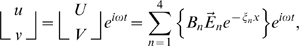
(11)and
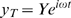



The four independent eigenvectors are 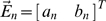, with corresponding eigenvalues *ξ_n_*. The coefficients, *B_n_*, are found from the four boundary conditions below.

### Mechanical boundary conditions

To model the isometric case, we require zero displacement at the ends of the stereocilia
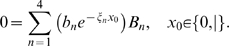
(12)To model the “zero force” case, we set the applied force to zero and require the axial force to balance the transverse force via the change in tension in the tip links to obtain
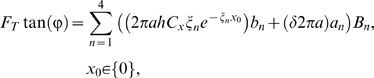
(13)In this model, the staircase structure and angled tip links are critical to coupling flexoelectric changes in stereocilium length to transverse hair bundle motion. In the absence of tip links 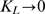, or in the absence of staircase structure, the angle 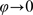, rendering *F_T_* = 0 in either case.

### Electrical conditions

In most simulations, we drive the stereocilia via a current injection at the tip, at the location of the MET channels. Under this condition, the voltage gradient is related to the current injection, *I_T_*, at the tip and the axial resistance per unit length, *r_i_*, according to *I_T_r_i_ = dV/dx*. Substitution into Eq. 11 gives
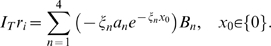
(14)The magnitude of the current entering the soma will be dependent on the somatic impedance. We consider two extreme conditions, the first having infinite somatic impedance (*I = 0*) and the second having zero somatic impedance (*V = 0*). These two conditions provide boundary conditions for infinite somatic impedance
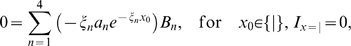
(15)and for zero somatic impedance
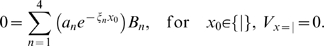
(16)Equations 12–16 were combined to solve for the complex-valued constants *{B_1_,B_2_,B_3_,B_4_}* and transverse displacement *Y*.

### Power Efficiency

Equations were solved with a sinusoidal input current under two conditions: zero-displacement (isometric, condition 0) and zero force (condition 1). Since the system is linear, the maximum power transfer to the mechanical load occurs when the load is matched to the stereocilia. This occurs approximately half way between the isometric and zero-force extremes and was used to determine the length at which the power transfer is most efficient. By superposition the efficiency 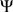 is equal to the mechanical power output divided by the electrical power input.
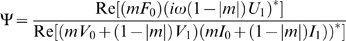
(17)where *F* is the force, *U* is the velocity, *V* is the voltage, *I* is the current and the subscripts 0 and 1 denote the isometric and zero-force cases, respectively. The complex-valued superposition parameter, *m*, was optimized to align the phase of the force with the velocity and maximize the power output. As expected, the efficiency depends upon the electrical admittance of the hair cell soma. To investigate this we considered the two extremes of “infinite” somatic impedance (zero current exiting the base) and “zero” somatic impedance (zero voltage modulation at the stereocilia base).

### Parameter Estimations

Physical parameters used in the present simulations are provided in Table 2. Aside from the geometry, which is known, there are only four key physical parameters: 1) fluid viscosity [41], [42], 2) the flexoelectric coefficient of lipid membranes [43] 3) the intracellular electrical conductance [37], and 4) the axial mechanical stiffness. Results are not very sensitive to other parameters. Therefore, axial stiffness is the only key physical parameter that has not been measured directly. In the model, this is the axial stiffness that is felt by the plasma membrane as it caps the axial actin core and, hypothetically, is dominated by actin polymerization/depolymerization dynamics within the stereocilia in addition to passive mechanics. The stiffness, although not yet directly measured, was selected here by matching the flexoelectric efficiency and best frequency (Fig. 4). Increasing the stiffness moves the solid cure to the right and decreasing the stiffness moves the curve to the left.

**Table 2:** Nominal Physical Parameters.

Symbol	Value	Description
*a*	1.6e-7	Stereocilia radius (m) [52], [53]
*a_b_*	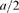	Stereocilia radius at base insertion to cell (m) [47]
*A_i_*	1e-15	Axial conductance cross-section (m^2^) (0.53*π*a ^2^)
*1/C_x_*	2.8e-4	Axial compliance re: *h* (m^2^/N) *(see text)*
*E_c_*	1e7	Transverse Young's modulus for bending (N/m^2^) [40]
*ε*	1e-11	Electrical permittivity (F/m) (ε/h = 1 µF/cm^2^, [37])
*f*	1.5e-18	Flexoelectric coefficient (N-m/V) [43]
φ	π/4	Tip-link angle (rad.) [54], [55]
*g_m_*	10	Membrane conductance (S/m^2^) [56]
*h*	1e-9	Membrane thickness (m) (ε/h = 1 µF/cm^2^, [37])
*K_L_*	1.5e-5	Tip link stiffness (N/m) [57]
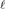	6e-6	Stereocilium length (m) *(see * *Fig. 4* *)*
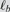	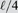	Stereocilium length of tapered section (m) [40]
*μ_0_*	1e-3	Fluid viscosity (N-s/m^2^) [41], [42],
*η*	0.7	Fractional visco-elastic power ( 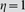 fluid, 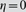 solid) [58]
*r_i_*	1.9e13	Axial resistance per unit length (Ohm/m) (σ*A_i_)^−1^
*ρ*	1e3	Fluid density (kg/m^3^) [42]
*σ_i_*	1.2	Intracellular conductivity (S-m/m^2^) [37]
*ω*		Stimulus frequency (rad/s)
